# Continuous diphenhydramine infusion and imatinib for KIT-D816V-negative mast cell activation syndrome: a case report

**DOI:** 10.1186/s13256-017-1278-3

**Published:** 2017-04-24

**Authors:** Faizan Malik, Naveed Ali, Syed Imran Mustafa Jafri, Ali Ghani, Mohsin Hamid, Margot Boigon, Christian Fidler

**Affiliations:** 1 Department of Internal Medicine, Abington Jefferson Health, 1200 Old York Road, Abington, PA 19001 USA; 2 Rosenfeld Cancer Center, Abington Jefferson Health, 1200 Old York Road, Abington, PA 19001 USA

**Keywords:** Mastocytosis, Mast cell activation disease, Mast cell activation syndrome, Continuous diphenhydramine infusion, Imatinib, Paucity of controlled trials

## Abstract

**Background:**

We present the first full case report of the treatment of mast cell activation syndrome with continuous diphenhydramine infusion, which resulted in the improvement of anaphylactic reactions and a decrease in hospital readmission. Furthermore, the patient received imatinib in the absence of the KIT-D816V mutation, which led to further improvement of quality of life. Currently, we are trying to wean this patient off diphenhydramine; if successful, this attempt will represent the first reported case.

**Case presentation:**

An 18-year-old white girl presented with a flare of mast cell activation syndrome and received epinephrine and steroids. She had failed multiple previous therapies, and her quality of life was affected due to two to three flares/week. She was started on continuous diphenhydramine infusion and imatinib, which led to a decrease in hospital admissions and marked improvement in her quality of life.

**Conclusions:**

Continuous diphenhydramine infusion can provide promising outcomes following the failure of intermittent antihistamine dosing in patients with severe mast cell activation syndrome. Initiating continuous diphenhydramine infusion may be helpful in an intensive care setting when the patient is particularly prone to anaphylaxis and/or the resources needed to manage anaphylaxis are not available outside the intensive care unit. Furthermore, imatinib provides benefits in KIT-D816V-negative mast cell disorders due to other unknown mutations.

## Background

Mast cell activation syndrome (MCAS) is characterized by recurrent symptoms of mast cell activation in combination with objective evidence of mast cell mediator release. The clinical features include flushing, pruritus, abdominal pain, diarrhea, hypotension, syncope, and musculoskeletal pain. The treatment options are H1 and H2 antihistamines, doxepin, cromolyn, and anti-leukotriene agents. The treatment options for refractory disease are based mainly on observational studies and case reports. More guidance from larger randomized controlled trials is needed, but in the absence of this guidance, clinicians use the available data in conjunction with their clinical expertise in the best interest of the patients. Recently, omalizumab has shown some efficacy in case reports. Maintenance steroids can be used in refractory cases but result in multiple side effects. We present the first full report of a case of MCAS treated with continuous diphenhydramine infusion (CDI), which resulted in the improvement of anaphylactic reactions and a decrease in hospital readmission. Furthermore, our patient received imatinib in the absence of the KIT-D816V mutation, which led to further improvement in her quality of life. Currently, we are trying to wean her off diphenhydramine; if successful, this attempt will be the first reported case.

## Case presentation

An 18-year-old white girl with a history of MCAS presented with flushing, diarrhea, and hypotension. She had chronic swelling of her face and abdomen at baseline. She was initially stabilized by epinephrine and steroids. She experienced multiple prior episodes of anaphylactic reactions at least twice weekly for which she was evaluated in our emergency room and treated per a standard protocol in place for her given the frequency of her visits.

At age 15, she had significant asthma symptoms and was found to have a mildly elevated immunoglobulin E (IgE) level. Prior skin testing resulted in vomiting, dehydration, and hospitalization. She was diagnosed as having food protein-induced enterocolitis with severe anaphylaxis to eggs after undergoing a food challenge test. A bone marrow biopsy was inconclusive without evidence of mastocytosis and the KIT-D816V mutation. Her serum tryptase level was normal between episodes and was elevated to 26.5 ng/ml during flare ups. Her plasma prostaglandin-D2 and 24-hour urine 11 betaprostaglandin-F2 levels were markedly elevated during episodes. A C1 esterase inhibitor test was negative.

She was previously treated with steroids, multiple H1 and H2 antihistamines (diphenhydramine 300 mg daily, loratadine 10 mg daily, cetirizine 10 mg daily, fexofenadine 120 mg daily, and famotidine 80 mg daily), omalizumab (one-time dose of 300 mg), and a monoclonal antibody that inhibited IgE binding to mast cells, which unfortunately also resulted in anaphylaxis. To stress the seriousness of the situation, she had a severe anaphylactic reaction resulting in cardiac arrest with successful resuscitation.

Of interest, the University of Minnesota recommended the use of CDI based on ongoing research that had shown some efficacy. She was admitted to our intensive care unit (ICU) and started on a diphenhydramine infusion at 5 mg/hour. Then, the infusion was increased by 2 mg every 2 hours with a target goal of 15 mg/hour. This approach led to a decrease in the episode frequency to one to two times a month, which felt like a miracle to the patient. Based on ongoing research, we started imatinib at 100 mg once daily and titrated up to 400 mg once daily, because this treatment had evidence of use in patients negative for the KIT-D816V mutation. This approach led to a further increase in intervals between episodes to once every 2 to 3 months. Therefore, our patient can enjoy months before an episode occurs, which is a new scenario for this patient in particular.

## Discussion

MCAS is an idiopathic mast cell disorder that is difficult to treat because no biomarkers predictive of effective therapeutics have been established. Many treatments have been shown to be helpful in various patients, but treatment in the absence of predictive biomarkers requires patience, persistence, and a methodical approach on the parts of both the patient and the physician when stepping through treatment trials until treatments that are significantly helpful are identified. Diphenhydramine has always been administered as intravenous pushes and only recently has been reported for use as an infusion for MCAS. We present a case of the success of this treatment, which has promising future outcomes.

Mast cells were discovered in 1863, and systemic organ involvement concomitant with skin disease was first described in 1949 [1]. Mast cell activation disease (MCAD) comprises the full spectrum of systemic mast cell disorders and can present synchronously with myeloproliferative syndrome [2], myelodysplastic syndrome [2], acute myeloid leukemia [3], or mast cell leukemia [4]. Mast cells release mediators, such as histamine, leukotrienes, proteases, or heparin, which lead to a broad spectrum of disease severity ranging from indolent to life-threatening [5]. The symptoms include pruritus, urticaria, bronchoconstriction, abdominal pain, diarrhea, recurrent syncope, flushing, and anaphylaxis [5]. The diagnosis of mastocytosis usually requires a tissue sample and histology, but the MCAS diagnosis is usually based on biochemical assessments of blood and urine that seek to identify elevated levels of mediators relatively specific to mast cells [6]. Systemic mastocytosis is mostly diagnosed by bone marrow biopsy to search for a multifocal, dense, and sharply demarcated infiltrate [7]. The serum tryptase level is a specific test for MCAS; an increase in this level above 15 ng/ml (upper limit of normal) in the presence of relevant symptoms is very helpful for the diagnosis [8]. The c-*Kit* (gain of function) mutation at codon 816 is present in most but not all adult onset systemic mastocytosis cases [9]. CD117 is a normal mast cell surface protein, whereas CD2 and CD25 are only present on the surfaces of abnormal mast cells. These markers are identified using multicolor flow cytometry or immunohistochemical staining techniques [10].

MCAS is defined by a constellation of clinical complaints attributable to pathologically increased mast cell activity, a focal or disseminated increase in mast cells, abnormal spindle shape in >25% of marrow mast cells, CD2 +/− CD25 expression, detection of genetic changes, tryptase in blood, N-methylhistamine in urine, heparin in blood, and a symptomatic response to mast cell inhibitors. A combination of three of these factors results in a diagnosis. The treatment mostly comes from anecdotal data or very small clinical trials performed for systemic mastocytosis due to the rarity of the disease and the lack of trials for MCAS. A cure is impossible due to the disorder’s genetic roots, and the treatment options are mostly directed at symptom management [11].

The first step is to identify and avoid the patient’s specific triggers. Due to the allergic nature of the disease, antihistamine H1 and H2 blockers, either alone or in combination, have long been used in the initial treatment of MCAS [12]. Steroids are also helpful and decrease the number of mast cells in the involved organs [13]. The addition of interferon-alfa to corticosteroids can further improve the symptoms, although this improvement obviously comes at the cost of serious side effects, such as myelosuppression and depression [14]. To avoid the side effects from steroids, steroid-sparing agents, such as azathioprine, or other immunosuppressive therapies, such as cyclosporine and methotrexate, should be considered [15].

MCAS can be life-threatening if it leads to anaphylaxis, which calls for alternate measures, such as venom immunotherapy [16]. The patient should always be in possession of an EpiPen (epinephrine autoinjector) in the case of an emergency. Our patient failed most of the abovementioned drugs.

Recently, omalizumab was tested as an experimental drug in four patients with a good clinical response. Two patients responded completely, one improved slowly, and one had an allergic reaction to the drug itself [17]. Unfortunately, our patient had an anaphylactic reaction to this drug, which further limited her options. These patients tend to be allergic to many medications, especially the excipients, such as fillers, binders, and preservatives.

The University of Minnesota studied ten patients with a disabling symptom frequency and placed them on CDI, resulting in marked improvement of symptoms and a downturn of flare frequencies [18]. The half-life of diphenhydramine is 1 hour, and intermittent dosing results in the cumbersome levels falling to subtherapeutic levels, thereby leading to another flare. The recommended dose is 10 to 14.5 mg/hour. Initiating CDI in an intensive care setting may be a helpful approach when the patient is particularly prone to anaphylaxis and/or the resources needed to manage anaphylaxis are not available outside the intensive care unit (ICU). We closely observed our patient in the ICU for 4 days. We started diphenhydramine at 5 mg/hour and then slowly increased the dose to achieve her target, which was 15 mg/hour. She had multiple episodes of skin urticaria, abdominal cramps, and breathing difficulties during her stay due to the disease itself. She also had nausea and vomiting, probably due to the excipients (preservatives) in the CDI preparation. The diphenhydramine most likely helped control the dysfunctional mast cells, whereas some excipient simultaneously aggravated the dysfunctional mast cells. She was discharged home after successful remission. The limitation of this technique is being attached to a pump 24/7, but this treatment is a gift for a patient who fights life every day and spends more time in hospitals than at home. This issue should be addressed using the shared decision-making technique.

The use of imatinib for mastocytosis was studied in a small clinical trial with 14 patients that proved successful. This trial included patients with the KIT-D816V mutation, *FIP1L1-PDGFR-alpha* rearrangement gene, and no mutation at all. Most of the patients responded to the drug [19]. Imatinib can provide benefits in some KIT-D816V-negative mast cell disorders because this drug “fits” well into the adenosine triphosphate (ATP)-binding pocket in KIT (where codon 816 is located), which prevents ATP from binding and activating KIT and the numerous pathways downstream from activated KIT. The KIT-D816V mutation not only prevents imatinib from binding at that site but also causes constitutive activation of KIT. Thus, imatinib usually is ineffective in controlling KIT-D816V-positive mast cell disease. Nevertheless, imatinib can benefit occasional patients with KIT-D816V-positive mast cell disorders, because some of those patients bear other activating mutations (that is, certain juxtamembrane mutations), which can also be inhibited by imatinib. Citing this data, we placed our patient on imatinib, which further decreased the frequency of flares and maintained her remission for months (to date). In the abovementioned study, none of the patients were taken off diphenhydramine infusion. Other kinase inhibitors, including dasatinib and nilotinib, have possible uses; theoretically, multitargeted kinase inhibitors, such as sunitinib and midostaurin, may be better than single targeted kinase inhibitors due to causative mutations in multiple genes [20]. However, these theories must be validated through clinical trials.

Montelukast can be used in patients with refractory abdominal and urinary symptoms [21]. Cladribine can reduce tryptase levels, bone marrow mast cell infiltrates, and the burden of KIT D816V+ mast cells but should be saved as a last resort due to mixed data and significant side effects [22]. Polychemotherapy followed by allogeneic stem cell transplant for advanced systemic mastocytosis was analyzed in a retrospective study of 40 patients and showed promising results; however, this method has not been studied in patients with MCAS to date [23]. Thalidomide, leflunomide, and many other drugs are still undergoing experimental testing and may be available in the future for the treatment of MCAS.

## Conclusions

MCAS is a common and potentially disabling disease. No randomized controlled trials have been performed using patients with this disease, and the therapeutic data are all anecdotal. All such cases should be reported to increase awareness about possible therapeutics. We provided novel therapies for our patient that led to a promising outcome.
